# Hyperandrogenism and Its Possible Effects on Endometrial Receptivity: A Review

**DOI:** 10.3390/ijms241512026

**Published:** 2023-07-27

**Authors:** Allia Najmie Muhammad Yusuf, Mohd Fariz Amri, Azizah Ugusman, Adila A. Hamid, Norhazlina Abdul Wahab, Mohd Helmy Mokhtar

**Affiliations:** 1 Department of Physiology, Faculty of Medicine, Universiti Kebangsaan Malaysia, Kuala Lumpur 56000, Malaysia; 2 Department of Biomedical Sciences, Faculty of Medicine and Health Sciences, University Malaysia Sabah, Kota Kinabalu 88400, Malaysia; 3 Department of Pathology, Faculty of Medicine and Health Sciences, University Malaysia Sabah, Kota Kinabalu 88400, Malaysia

**Keywords:** hyperandrogenism, endometrial receptivity, androgen excess, infertility

## Abstract

Endometrial receptivity is a state of the endometrium defined by its readiness for embryo implantation. When the receptivity of the endometrium is impaired due to hyperandrogenism or androgen excess, this condition can lead to pregnancy loss or infertility. Hyperandrogenism encompasses a wide range of clinical manifestations, including polycystic ovary syndrome (PCOS), idiopathic hirsutism, hirsutism and hyperandrogaenemia, non-classical congenital adrenal hyperplasia, hyperandrogenism, insulin resistance, acanthosis nigricans (HAIR-AN), ovarian or adrenal androgen-secreting neoplasms, Cushing’s syndrome, and hyperprolactinaemia. Recurrent miscarriages have been shown to be closely related to elevated testosterone levels, which alter the endometrial milieu so that it is less favourable for embryo implantation. There are mechanisms for endometrial receptivity that are affected by excess androgen. The HOXA gene, aVβ3 integrin, CDK signalling pathway, MECA-79, and MAGEA-11 were the genes and proteins affect endometrial receptivity in the presence of a hyperandrogenic state. In this review, we would like to explore the other manifestations of androgen excess focusing on causes other than PCOS and learn possible mechanisms of endometrial receptivity behind androgen excess leading to pregnancy loss or infertility.

## 1. Introduction

Infertility studies conducted in 190 countries between 1990 and 2010 found 48.5 million infertile couples, of whom 19.2 million had primary infertility problems and 29.3 million had secondary infertility problems [1]. In about 15% of infertile couples, the underlying cause cannot be determined [2]. Understanding endometrial receptivity is fundamental to understand unexplained infertility and pregnancy loss. Recently, a study has shown that endometrial receptivity is altered in patients with polycystic ovary syndrome (PCOS), suggesting that hyperandrogenism, along with insulin resistance and obesity, is an explanation for poorer embryo implantation and pregnancy outcome [3]. Numerous publications have highlighted the association between hyperandrogenism and infertility in PCOS patients. However, in this review, we would like to explore the other phenotypes of androgen excess in addition to PCOS and learn of possible mechanisms of endometrial receptivity behind androgen excess leading to pregnancy loss or infertility.

### 1.1. Normal Androgen Physiology

Physiologically, androgens in women are produced in the ovaries, adrenal glands and peripheral tissues. Under normal circumstances, the ovaries and adrenal glands contribute approximately equally to testosterone production. Almost all testosterone is produced by direct testosterone secretion from the adrenal glands. A small portion of testosterone production is formed by peripheral conversion of circulating androstenedione, which is secreted into tissues mainly from the ovaries by the enzyme 17-β-hydroxysteroid dehydrogenase (HSD) [4]. 

Testosterone, dehydroepiandrosterone sulphate (DHEAS), dehydroepiandrosterone (DHEA), androstenedione, and androstenediol are the androgens released by the endocrine glands [5]. Androstenedione, which is synthesised in the ovaries and adrenal glands, is a direct precursor of testosterone [5]. DHEAS and DHEA are also precursors of testosterone [6]. DHEAS (produced in the adrenal glands) and DHEA (produced in both the ovaries and adrenal glands) are converted into androstenedione, which is then converted into testosterone. DHEAS is an essential measure of adrenal androgen production because it is produced exclusively in the adrenal glands [5]. These androgens are secreted in response to luteinising hormone (LH) in the ovaries and adrenocorticotropic hormone (ACTH) in the adrenal glands.

**Figure 1:**
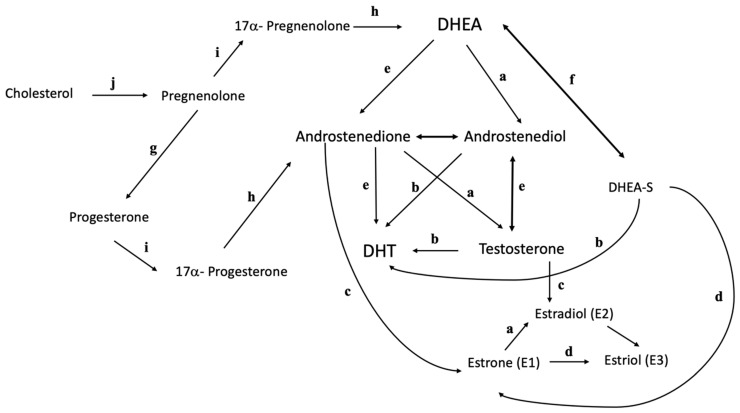
Interconversion of testosterone and testosterone precursors. a, 17-β-hydroxysteroid dehydrogenase; b, 5α-reductase; c, P450 aromatase; d, 16α- hydroxylase; e, 3β-hydroxysteroid dehydrogenase; f, DHEA sulfotransferase; g, 3β-hydroxysteroid dehydrogenase type 1; h, 17, 20 lyase; i, 17α-hydroxylase; j, cholesterol side chain cleavage.

In addition to the ovaries and adrenal glands, androgens are also produced by the peripheral conversion of prohormones of adrenal and ovarian origin in non-endocrine tissues. In women, androstenedione is the predominant precursor of serum testosterone, making it the most important prohormone [7]. In addition, 5-alpha-reductase, found in the liver, hair follicles, and other androgenic target cells, convert testosterone into dihydrotestosterone (DHT), a potent androgen [8]. Figure 1 below shows a summary of the interconversion of testosterone and testosterone precursors [9]. 

Although the sites of androgen synthesis are clearly known, the regulation of androgen production is unclear. Currently, testosterone secretion is thought to be a by-product of oestrogen production in the ovaries and glucocorticoid production in the adrenal glands. It has been shown that androgen secretion in the adrenal glands is related to factors that influence cortisol secretion in addition to ACTH levels. Thus, an increase in cortisol and ACTH leads to an increase in serum androgens [7,10]. In contrast, the control of hypothalamic, pituitary, and ovarian hormones is more complex, with oestradiol and progesterone playing an important role in the regulation of gonadotropins.

Metabolic clearance of androgens also plays an important role in regulating serum testosterone levels. In addition to increased production of androgens, decreased androgen clearance results in increased serum androgen levels. Conditions associated with decreased androgen clearance include oestrogen treatment [4,11], barbiturate treatment [4,12], hyperthyroidism [4,13], hypogonadism [4,14], and ageing [15]. 

Besides oestrogen and progesterone, androgen also plays an important role in the menstrual cycle. During the oestrogen-dominated proliferative phase, stromal fibroblasts in the functional (upper) layer of the human endometrium exhibit the highest expression of AR [16], which is down-regulated during the secretory phase but is maintained in stromal cells within the basal compartment throughout the cycle [17,18]. Expression of AR is also increased in glandular epithelial cells and is up-regulated during the mid-secretory phase [19]. Furthermore, expression of AR has been detected in both stromal and epithelial cells in the first trimester decidua [19]. In contrast, AR has been identified in the perivascular stromal cells in the functional layer, but not in the endothelial cells [6]. With this knowledge, we can assume that the effects of progestins and androgens are mediated indirectly by perivascular stromal cells, whereas the direct effects of oestrogens on endometrial vasculature, including angiogenesis and permeability, are likely to be mediated by the oestrogen receptor (ER).

In the context of endometrial receptivity, androgen receptor (AR) expression is restricted to the stroma of the endometrium and changes during the menstrual cycle, gradually decreasing from the early proliferative to the mid-secretory phase [20]. Regulation of uterine androgen receptors (AR) contributes to the normal pregnancy process. This reduction has been found to correlate with significant differential expressions of the *Spp1*, *Prl*, *Igfbp1*, and *Hbegf* genes that are associated with endometrial receptivity and endometrial decidualisation [21]. Hyperandrogenism leads to increased expression of AR and implantation failure due to aberrant expression of genes related to implantation and mitochondrial function.

### 1.2. Hyperandrogenism

In a healthy woman, androgen is secreted in almost equal amounts by the ovaries or the adrenal gland. Despite intensive research, however, we know of only a few causes of androgen excess in women. In order to better understand and classify hyperandrogenism, the source of the androgen excess must be determined.

The source of hyperandrogenism may be the ovaries and the adrenal glands. Typically, androgen secreted by the adrenal gland predominates in non-classical congenital adrenal hyperplasia (NC-CAH), while the ovary is the main source of androgens in polycystic ovary syndrome (PCOS) and idiopathic hyperandrogenism [22,23]. However, previous data suggest that 35% of PCOS cases, 50% of idiopathic hyperandrogenism cases, and about 70% of patients with NC-CAH have excess androgens from more than one source [24]. A more detailed explanation of the non-PCOS causes is discussed in the next section.

The primary source of androgens in PCOS, the most common disorder with androgen excess, is the ovaries. Ovarian hyperandrogenism is the primary pathogenetic mechanism for the syndrome, although no genetic impairment of ovarian enzymes has been identified [25]. In addition, both increased primary androgen secretion by the theca cells and increased drive by luteinising hormone (LH) (and insulin) contribute to increased ovarian androstenedione and testosterone production [26,27]. Theca cells from polycystic ovaries even produce more androstenedione both under basal conditions and during stimulation by gonadotrophins [26,27].

However, in many women with PCOS, there are multiple causes of androgen hypersecretion. About 50% of women with PCOS have elevated circulating levels of dehydroepiandrosterone sulphate (DHEAS) and 11β-hydroxyandrostenedione, two androgens secreted almost exclusively by the zona reticularis of the adrenal glands [28,29]. The elevated DHEAS levels in women with PCOS are likely due to the cumulative effect of several factors, including increased circulating unbound oestradiol levels and altered cortisol metabolism [30,31]. Indeed, decreased peripheral cortisol has been observed in PCOS, due to increased inactivation of this steroid by 5-alpha-reductase or impaired reactivation of cortisol from cortisone by 11beta-hydroxysteroid dehydrogenase type 1 [32,33]. Therefore, the decrease in peripheral cortisol would lead to decreased negative feedback on ACTH, so that the activity of the pituitary–adrenal–androgen axis would increase to maintain normal cortisol levels.

Elevated insulin levels are also thought to be the main cause of increased adrenal androgen secretion in women with PCOS [34]. However, serum DHEAS levels are typically lower in obese women with PCOS [35], and serum DHEAS correlates negatively with serum insulin in hyperandrogenic women [24]. Therefore, it is unlikely that hyperinsulinemia is the primary cause of elevated DHEAS levels. However, this does not rule out a role for insulin in adrenal androgen excess in women with PCOS.

In general, the source of androgen is not thought to affect the phenotype of diseases with androgen excess. While there are many differences in phenotype between the various androgen disorders, particularly between severe (PCOS) and mild syndromes [36], these differences appear to be determined primarily by other features of the syndrome (mainly insulin resistance). However, regardless of the type of androgen excess disorder (PCOS or idiopathic hyperandrogenism), hyperandrogenic patients with elevated DHEAS tend to be leaner, and have lower insulin levels and a better metabolic profile [36]. These findings have yet to be confirmed, but they raise the possibility that elevated DHEAS levels may have a protective effect on metabolic syndrome or that elevated insulin levels suppress DHEAS secretion [37].

## 2. Hyperandrogenic Syndromes

### 2.1. Androgen-Secreting Tumours 

Pure androgen-secreting adrenal tumours were found less frequently compared to other adrenal tumours. Most adrenal tumours are clinically silent, but when functional they usually secrete cortisol, aldosterone, or catecholamines. This leads to the symptoms of androgen-secreting tumours, which can be divided into three categories: hirsutism, virilisation, and menstrual cycle disorders [15]. On the other hand, only about 1% of ovarian tumours secrete androgens, resulting in clinical hyperandrogenism. The most common androgen-secreting ovarian tumour is the Sertoli–Leydig cell tumour, which accounts for about 0.5% of all ovarian tumours. In women of reproductive age, this tumour is usually benign and unilateral.

Androgen-secreting tumours are a rare cause of hirsutism. In more than half of the reported cases, the tumours proved to be malignant and may be of ovarian or adrenal origin [38]. The typical presentation is hirsutism (excessive hair growth in androgen-sensitive areas such as the face, chest, nipples, buttocks, and external genitalia). Increasing musculature, a deeper voice, breast atrophy, male pattern baldness, and clitoromegaly are signs of virilisation. [15,38]. 

Physical examination may indicate abdominal or pelvic masses; if these originate from the adrenal gland, there is often concomitant hypercortisolaemia and elevated levels of dehydroepiandrosterone and dehydroepiandrosterone sulphate (DHEAS), leading to Cushing’s syndrome [38]. In a patient with suspected hyperandrogenism associated with an androgen-secreting tumour, a thorough examination should be performed to assess the severity, timing of onset, and progression of symptoms. In postmenopausal women with an unknown cause of increased testosterone and virilisation, the suspicion of an androgen-secreting tumour should be suspected, whether it is ovarian or adrenal in origin [39].

### 2.2. Congenital Adrenal Hyperplasia

Congenital adrenal hyperplasia (CAH) is inherited in an autosomal recessive manner and disrupts adrenal steroidogenesis [40,41]. It is caused by the absence of one of the enzymes involved in the synthesis of adrenal steroid hormones, usually a deficiency of 21-hydroxylase, which diverts the precursors into the androgen pathway [41,42]. The specific mutation of the genes involved in the disruption of adrenal steroidogenesis results in different clinical features. In 21-hydroxylase deficiency, the clinical spectrum ranges from salt-wasting to simple or mild virilisation.

In classical CAH (CCAH), features of both salt wasting and simple virilisation are observed [40]. The mild variant is called non-classical CAH (NC-CAH). CAH is characterised by cortisol deficiency, with or without aldosterone deficiency, and androgen excess [41]. Physiologically, the hypothalamic–pituitary–adrenal (HPA) axis regulates cortisol secretion from the adrenal cortex. Corticotrophin-releasing hormone (CRH) produced by the hypothalamus regulates the release of adrenocorticotrophic hormone (ACTH), which subsequently stimulates the adrenal cortex. When the adrenal gland releases cortisol, a negative feedback mechanism signals the hypothalamus to regulate the secretion of CRH and ACTH [42].

In CCAH, impaired cortisol synthesis leads to loss of negative feedback inhibition of cortisol, an increase in hypothalamic CRH, and an increase in ACTH secretion in the pituitary. The excessive ACTH secretion leads to an accumulation of cortisol. The hyperandrogenic element associated with these enzyme deficiency disorders is the result of altered metabolism of cortisol [40]. Decreased enzymatic activity of 21-hydroxylase impairs the biosynthesis of cortisol, leading to an increase in the concentration of 17-hydroxyprogesterone (17-OHP) and progesterone. This leads to poor cardiac function, increased secretion of antidiuretic hormone (ADH), and exacerbated mineralocorticoid deficiency in affected individuals [43]. 

In CAH, the concentration of the substrates in close proximity to 21-hydroxylase, progesterone and 17-OHP, is increased due to the deficiency of 21-hydroxylase. In addition, the defective proteins are confined to the adrenal cortex. The androgen receptor in the adrenal cortex affects steroid metabolism and response to steroids. Apart from this, an increase in the concentration of dihydrotestosterone, DHT (17-OHP, which is converted to DHT by 5α-reductase) from alternative pathways of steroidogenesis further aggravates the symptoms of androgen excess. This pathway is probably the cause of the prenatal virilisation seen in affected female foetuses due to androgen excess [40].

In affected infants, CCAH usually occurs in the neonatal period. Symptoms vary depending on the sex of the infants [40]. Diagnosis of the disease is difficult, especially in affected neonates, who are at higher risk of hyponatraemia, hypokalaemia, and hypotension with an unknown, possibly fatal, outcome [44]. Prenatal virilisation also makes it difficult to determine the “true” sex of the newborn at birth. In most cases, they have ambiguous genitalia [40]. Therefore, a higher index of suspicion for 21-OHD is required to be excluded in this case.

Children with NC-CAH often show premature puberty (premature development of pubic hair, axillary hair, or apocrine odour before the age of 8 or 9 in girls and boys, respectively). Girls may have the enlargement of the clitoris, while boys may have phallic enlargement with testes of prepubertal size. Although premature puberty is common in children with CAH, it is a rare cause of premature adrenarche [45]. CAH is diagnosed when testosterone, 17-OHP, and androstenedione are elevated, with or without advanced bone age [40,46].

Common symptoms in female adolescents and adults with NC-CAH include irregular menstruation, chronic anovulation, infertility, acne, and hirsutism, which is reported as the most common symptom [47,48,49,50]. Distinguishing between NC-CAH and polycystic ovary syndrome (PCOS) is quite difficult due to the similarity of symptoms [43,51]. It has been shown that women with NC-CAH are more likely to have elevated 17-OHP and progesterone levels compared to women with PCOS [52]. Women with PCOS usually have oligomenorrhoea, biochemical or clinical signs of hyperandrogenism, obesity, insulin resistance, polycystic ovarian morphology, and an increased LH/FSH ratio. However, even with these features, it is difficult to distinguish women suffering from NC-CAH from PCOS [53]. In a previous study, it was reported that the women on NC-CAH did not suffer from symptoms of androgen excess. They achieved normal body size without adrenal insufficiency and also suffered from infertility [54].

As mentioned earlier, women with NC-CAH have many similarities with PCOS individuals. One mechanism of PCOS is disruption of the hypothalamic-pituitary-ovarian (HPO) axis, which can also occur in individuals with NC-CAH. It has been postulated that androgen overexposure in the uterus leads to changes in the mechanisms that control GnRH and kisspeptin. In another study, it was found that an increase in LH concentrations with an increase in LH pulse amplitude was highly associated with high androgen concentrations in women with NC-CAH [55,56].

Ovarian function can be directly influenced by androgens. The occurrence of polycystic ovarian morphology is highly influenced by excessive androgens, which may arise either from endogenous androgens as in CAH or from exogenous androgens as in illicit steroid abuse in female-to-male transgender or female athletes [56,57]. Androgen receptors are expressed in granulosa cells, theca cells, and oocytes [58]. A study using tissue-specific androgen receptor knockout mice revealed that androgens regulate follicular growth via androgen receptors at different stages of follicular development [59,60]. Physiologically, the function of androgens is to promote the initial growth of small antral follicles. However, hyperandrogenism leads to follicular arrest and the inability to select the dominant follicle [61]. Androgens can stimulate the extracellular matrix, leading to stromal hyperplasia. Thus, disruption of the HPO axis by androgens can occur at multiple sites [62].

Excess circulating androgens and progesterone may impair HPO axis function and endometrial receptivity. Increased progesterone concentrations impair the quality of cervical mucus, accelerate endometrial maturation, reduce endometrial receptivity, decrease sperm penetration, and impair embryo implantation [63]. Therefore, to ensure ovulation, proliferation of endometrium and implantation of the embryo, adequate suppression of progesterone (<60 ng/dL) and plasma renin activity is required in both CCAH and NC-CAH. Fertility rates are higher when glucocorticoid therapy is administered [50].

### 2.3. Hyperandrogenic Insulin-Resistant Acanthosis Nigricans (HAIR-AN) Syndrome

Hyperandrogenic insulin-resistant acanthosis nigricans (HAIR-AN) is a subtype of PCOS characterised by high insulin resistance [64]. Obesity, genetic, and environmental variables are associated with the development of HAIR-AN. Diagnosis is predominantly clinical, with laboratory studies providing further support. HAIR-AN syndrome is observed in 1 to 3 percent of hyperandrogenic women [45]. When triggered by LH or HCG, ovarian stromal cells synthesise androgens, depending on the pathophysiology. It has also been found to increase the steroidogenic activity of these cells. The latter is an important factor in determining the extent of hirsutism. There may be a closer relationship between the extent of hirsutism and the degree of hyperandrogenism observed. Insulin-like growth factor-1 (IGF1), a proteic peptide with close similarity to insulin, has the same steroidogenesis capacity as the latter [65].

It has been reported that maternal history of metabolic syndrome and the presence of acanthosis nigricans may lead to insulin resistance and be responsible for hyperandrogenism and virilisation. The approach to reduce insulin resistance was lifestyle modification. Treatment to reduce ovarian hyperandrogenism, such as combined oestrogen and progestin pills, are used because of their antigonadal effect, which inhibits LH, leading to a reduction in ovarian androgens; their increase in sex hormone-binding globulin is also known to reduce bioavailable testosterone [65].

Genetic insulin resistance disorders of type A are characterised by hyperandrogenism, insulin resistance, and acanthosis nigricans (AN). Type B IR is caused by circulating antibodies directed against the insulin receptor and is associated with other autoimmune diseases [66,67]. The HAIR-AN syndrome occurs in 1 to 5% of young women with hyperandrogenism, mainly in young black African women. Because of its subtle symptoms, its prevalence may be underestimated. It is a rare syndrome that causes an unusual multisystem disorder in women and is misdiagnosed in many cases [66].

Excess circulating insulin leads to increased expression of insulin growth factor receptors (IGFR) on epidermal keratinocytes and melanocytes, leading to AN [68]. Circulating insulin can also activate ovarian stromal cells and granulosa cells to produce excess androgens, which are responsible for the symptoms of hyperandrogenism. In addition to AN, clinical examination reveals indicators of virilisation, such as hirsutism, android obesity, clitoral hypertrophy, muscle hypertrophy and increased desire. Other symptoms of hyperandrogenism, besides virilisation, are amenorrhoea, hypofertility or infertility, retention acne, and androgenetic alopecia [69,70].

### 2.4. Hirsutism

Hirsutism is the excessive development of terminal hair in females in a typically male pattern [36]. It is usually a symptom of high androgen levels. Hirsutism occurs in 5–15% of women and is often associated with a lower quality of life and severe psychological distress. Hirsutism should be distinguished from hypertrichosis, which is defined as increased development of vellus hair in a general, non-sex-specific distribution independent of androgens. However, hyperandrogenism may exacerbate the condition [49].

Hirsutism is a clinical diagnosis, and the prevalence varies according to the diagnostic criteria used. The modified Ferriman–Gallwey scoring system, which consists of nine androgen-sensitive body regions, is commonly used to diagnose hirsutism [71]. Cut-off scores vary by race and ethnicity: hirsutism is defined as a score of 8 or higher in black and white women in the United Kingdom and the United States, and a score of 9 or higher in Mediterranean women. Hispanic and Middle Eastern women have a score of 6 or higher; South American women have a score of 2 or higher, and Asian women have a score of 2 or higher. Scores between 15 and 25 indicate mild hirsutism, while scores above 25 indicate severe hirsutism. The subjective nature of this scoring method, and the fact that locally high scores or previous cosmetic treatments cannot be taken into account, are two of the limitations of this method. The Endocrine Society advocates the treatment of patient-relevant hirsutism, i.e., excessive hair growth in the genital area from which patients suffer [38].

Idiopathic hirsutism is a diagnosis of exclusion that occurs in approximately 10% of women with regular menstruation, normal ovarian morphology, and normal serum androgen levels. Data suggest that almost half of all women with mild hirsutism have “idiopathic hirsutism”.

## 3. Endometrial Receptivity

The term “endometrial receptivity” refers to the brief window of endometrial maturation in which the trophectoderm of the blastocyst can adhere to the epithelial cells of the endometrium before invading the endometrial stroma and vasculature [72]. Since Rock and Bartlett described the criteria for dating and the histological changes of the endometrium, the receptivity of the endometrium and the characteristics of the implantation window have been the subject of extensive research [73]. Although aberrant endometrial receptivity is usually investigated in infertility, it is actually responsible for a whole spectrum of reproductive diseases, ranging from complete failure to implant (infertility) to severe implantation failure (miscarriage) to mildly abnormal implantation and invasion (e.g., pre-eclampsia).

Most women become ready to conceive in the mid-luteal phase due to the sequential effects of the steroid hormones, namely oestrogen and progesterone. Both oestrogen and progesterone play an important role in the establishment of the endometrial transition, which promotes embryo implantation. This is evidenced by the compartmentalised expression of the oestrogen receptor (ER) and the progesterone receptor (PR) at different periods of peri-implantation, providing insight into where the coordinated actions of oestrogen and progesterone occur in preparing the uterus for implantation and decidualisation during early pregnancy [74].

Decidualisation is a necessary process for embryo implantation in which proliferating fibroblasts transform into specialised secretory cells responsible for producing factors that promote endometrial receptivity [75]. This process depends on activation of cyclic adenosine monophosphate (cAMP) and progesterone signalling pathways and inhibition of oestrogen signalling, resulting in decreased cellular proliferation and DNA synthesis, as well as a reduction in cellular mitotic activity [76]. Successful decidualisation leads to significant morphological and biochemical changes, including glandular secretion, extensive vascular remodelling, and an influx of natural killer (uNK) cells from the uterus [77].

Implantation of the embryo occurs in three phases, namely (i) apposition, (ii) adhesion, and (iii) invasion [78]. During apposition, the blastocyst juxtaposes with the endometrial implantation site rendering the blastocyst immobile and the embryo shows signs of polarity about 2–4 days after the morula enters the cavity. In the adhesion phase, the trophoblast cells of the blastocyst attach to the receptive endometrium, mediated by pinopodes of the luminal epithelium, adhesion molecules, and signalling factors. Thereafter, in the invasion phase, the invading trophoblast cells navigate through the endometrial basement membrane, invade the endometrial stroma and then migrate into the maternal decidua, bringing nutrients from the maternal circulation into the embryo [78,79]. During these phases, a multitude of molecular mediators plays a role in the initial maternal-foetal connection, which is regulated by ovarian steroid hormones. These mediators include lipids, cytokines, growth factors, adhesion molecules, and others [80,81]. 

Adequate maturation of the endometrium is vital for endometrial receptivity, which is led by oestrogen and progesterone. Oestrogen stimulates the basal layer of the endometrium to grow rapidly (proliferation phase) at both the glandular and stromal levels. By stimulating the stromal cells to produce growth factors such as IGF1 and epidermal growth factor (EGF), which interact with receptors produced by the epithelial tissue, oestrogen causes glandular proliferation [82]. After the corpus luteum has started to produce oestrogen and progesterone after ovulation, the endometrial glandular epithelium shows secretory changes. Progesterone, through its action on the endometrium, stimulates the active secretion of glycoproteins and peptides into the endometrial cavity and inhibits proliferation of the endometrial epithelium due to progesterone-mediated blockade of oestrogen receptor expression [83] as well as activation of 17β-hydroxysteroid dehydrogenase and sulfotransferase activity, which convert oestradiol to oestrone [84]. The peak of secretion activity occurs about 7 days after the luteinising hormone (LH) spike, the endometrial stroma becomes highly oedematous, and vascular proliferation occurs. Under the influence of progesterone, decidualisation occurs, characterised by increased mitosis and differentiation of stromal cells, as well as progesterone-dependent infiltration of specific leukocyte subsets into the endometrial stroma, including natural killer cells, T cells, and macrophages [84,85]. The decidualised stroma provides protection and supports conceptus implantation [82]. In the absence of fertilisation, the corpus luteum degenerates between the 24th and 28th day, resulting in a marked decrease in progesterone and the detachment of the stratum functionalis, which marks the beginning of the menstrual cycle. 

The primary morphological change that characterises endometrial receptivity is undoubtedly the presence of pinopodes, bleb-like structures of variable length that project into the uterine lumen just above the microvilli level of adjacent cells and are present on the apical surface of the endometrial epithelium [78,79]. Endometrial pinopodes are considered morphological markers of endometrial receptivity and are only present for a short period of time corresponding to the implantation window [86].

## 4. Assessment of Endometrial Receptivity

There are no specific tests to determine endometrial receptivity. However, some methods have been suggested, including the use of ultrasound, hysteroscopy inspection, and uterine natural killer cells, as well as endometrial receptivity array. 

Ultrasonography, particularly transvaginal ultrasonography, offers fairly reliable, non-invasive methods for assessing endometrial receptivity. Several ultrasound parameters and patterns have been proposed. The most commonly used parameter is endometrial thickness, i.e., the maximum distance between the endometrial–myometrial interface on the anterior and posterior uterine walls in a median longitudinal plane of the uterus. In contrast, the endometrial pattern is usually divided into three common patterns: the three-line pattern consisting of a central hyperechogenic line surrounded by two hypoechogenic layers, the intermediate isoechogenic pattern with the same reflectivity as the surrounding myometrium, and a poorly defined central echogenic line, a homogeneous hyperechogenic endometrium [87]. Endometrial thickness assessment with a cut-off of 6 mm had the highest sensitivity in predicting receptive endometrium [88]. The triple-line pattern, on the other hand, showed the highest accuracy in predicting receptive endometrium [88]. In assisted reproduction, endometrial thickness and pattern are usually determined on the day of human chorionic gonadotropin (hCG) administration [87]. In addition, an endometrial volume greater than 2 mL measured by ultrasound on the day of hCG injection is also useful for predicting receptive endometrium [89]. The use of Doppler to determine blood flow prior to hCG administration resulted in higher clinical pregnancy rates and implantation rates [88,90]. Although data were limited, a low frequency of uterine contractions (less than two contractions) 5 min after embryo transfer showed a significantly higher probability of clinical pregnancy [91].

Hysteroscopic examination of the uterus also helps to identify the receptive endometrium. The mid-luteal endometrium has been classified as ‘good’ during hysteroscopic assessment due to the ring type aspect of the glandular openings and the presence of well-developed varicose-like vessels [88].

The majority of white blood cells in the endometrium are uterine natural killer (NK) cells, whose numbers change according to the phase of the menstrual cycle. Their numbers increase dramatically when progesterone levels rise, particularly during the secretory phase [92]. Therefore, they have been extensively studied as a possible marker for the receptive endometrium. The role of uterine NK cells in reaching the receptive endometrium is in remodelling the maternal spiral artery as well as maintaining immune tolerance and suppressing inflammation by inhibiting TH17 [93,94]. The phenotype of uterine NK cells determined by immunohistochemistry is predominantly CD56^bright^CD16^−^, in contrast to the phenotype of peripheral NK cells of CD56^dim^CD16^+^. These CD56^bright^ NK cells, preferably uNK, produce cytokines and have little cytotoxicity, despite their abundant intracellular granules containing granzymes, granulysin, and perforin [95]. The maintenance of immune tolerance is crucial for the survival of the embryo in utero; however, the value of uterine NK cells in relation to endometrial receptivity is not yet well defined.

Advances in molecular testing led to the development of the Endometrial Receptivity Array (ERA), which determines endometrial receptivity by analysing endometrial biopsies for the expression of 238 selected genes with 134 transcriptomic signatures. These analyses classify the endometrium as receptive, pre-receptive, and proliferative [96]. The transcriptome reflects the actively expressed genes at each time point within the specific tissue types analysed. Thus, it allows a sample-specific molecular profile at the mRNA level, also known as a transcriptomic signature, to characterise tissue function. Therefore, the first study on gene expression profiles during the natural cycle to determine the transcriptomic signature as a basis for identifying the receptive endometrium was conducted by Mirkin et al. [97]. Since then, in another study, Diaz-Gimeno et al. subdivided the additional transcriptomic profile into late pre-receptive, optimal receptive, late receptive, and post-receptive using 238 sub-signatures. They also found that an optimal receptive signature was associated with a sustained pregnancy rate of 80%, and that a late receptive signature was associated with a 50% risk of biochemical pregnancy [98]. Since then, ERA has become the gold standard for diagnosing implantation windows in patients with implantation failure [99,100], as well as for investigating endometrial receptivity [101,102,103].

A meta-analysis of previous studies has shown that hyperandrogenism results in a significantly thinner endometrium compared to patients with normal androgen levels [104,105,106]. In addition, women with PCOS who have clinical or biochemical hyperandrogenism showed significant impaired blood flow to the subendometrium and endometrium, but there was no significant difference in endometrial volume in patients with hyperandrogenism [107]. 

Table 1 shows a summary of the methods used to determine endometrial receptivity and the associated parameters for receptive endometrium.

## 5. Mechanism of Hyperandrogenism Affecting Endometrial Receptivity

Androgens regulate endometrial receptivity by binding to androgen receptors (AR), which are widely distributed in glands, luminal epithelium, and stroma of the uterus [109]. Biologically, the conversion of androgens (testosterone and androstenedione) into oestrogens (oestrone and oestradiol) is catalysed by aromatase [110]. Aromatase belongs to the member of the cytochrome P450 family [111] and is produced by the CYP19 gene [112]. It has been found that a hyperandrogenic state (testosterone level of more than 2.44 nmol/L), especially in polycystic ovary syndrome (PCOS), significantly inhibits aromatase activity [113]. The HA-WT1 signalling pathway is also responsible for the impairment of endometrial receptivity. An increase in androgen receptors led to a decrease in the Wilms tumour suppressor gene (WT1). This decrease inhibits the epidermal growth factor receptor (EGFR) whose combination with its ligand EGF is associated with embryo implantation [109,114].

Hyperandrogenism and the resulting high expression of AR have been found to lead to defects in uterine cells due to aberrant expression of genes related to implantation and mitochondrial function. The resulting downstream effects of hyperandrogenism lead to mitochondrial dysfunction via increased expression of the protein for oxidative phosphorylation (OXPHOS) in the uterus in conjunction with low expression of *Nrf1* mRNA, as well as mitochondrial functional proteins VDAC, PHB1. The effects also extended to aberrant expression of implantation-related genes, where there is high expression of *Nr2f2*, *Ptch*, *Pgr*, and *Hbegf*, as well as low expression of *Spp1*, *Igfbp1*, *Hoxa11*, and *p21^WAF1/CIP1^ Hoxa11.* These aberrant expressions lead to abnormal implantation and ultimately compromise pregnancy outcomes [21].

In each reproductive cycle, genes from HOX are responsible for the growth, differentiation, and receptivity of the endometrium by mediating some sex steroid functions. Both HOXA10 and HOXA11 mRNA are found in the epithelial and stromal cells of the human endometrium, and their expression is significantly increased in the mid- and late secretory phases. This coincides with high levels of oestrogen and progesterone, which are favourable for embryo implantation [115,116]. Physiologically, HOXA-10 was increased in response to an increase in oestrogen and progesterone. However, increased androgen levels had the opposite effect. The expression of avβ3 integrin, HOXA-10, HOXA-11, and IGFBP-1 is decreased during the secretory phase in PCOS patients [115,116,117]. Testosterone caused a dose-dependent decrease in HOXA-10 mRNA demonstrated in vitro [117], suggesting a role for androgen reduction in improving endometrial receptivity. In PCOS, androgen receptors are highly expressed, resulting in the inability to down-regulate the oestrogen receptor-α in the implantation window [116,118]. In addition, overexpression of the steroid receptor coactivators AIB1 (Nuclear Receptor Coactivator 3) and TIF2 (Transcriptional Intermediary Factor 2) can enhance oestrogen activity in the endometrial cells of PCOS [118]. Dysregulation of steroid receptor expression and decreased expression of uterine receptivity markers and activity may contribute to the high infertility or recurrent miscarriages observed in women with PCOS. Remission of hyperandrogenism was achieved following improvement in HOXA-10 and HOXA-11 levels after laparoscopic ovarian drilling [119].

In addition, the androgen also influences the expression of various proteins in the endometrial cells. Hyperandrogenism leads to a reduction in CDKN2a which, in turn, impairs cyclin-dependent kinase (CDK) activity [109]. This inhibition of CDKN2a also leads to a significant decrease in the expression of CDKN2a protein in Ishikawa cells and a decrease in invasion, proliferation, as well as the rate of attachment of Jar spheroids to the Ishikawa cell monolayer [120]. This study suggests that supraphysiological androgen levels may affect the expression of proteins responsible for endometrial development and embryo implantation, which could be a cause of impaired endometrial receptivity and pregnancy loss [120]. Elevated testosterone has also been found to down-regulate the expression and distribution of L-selectin ligand (MECA-79) in the uterus, as well as reduce implantation sites [121]. 

Hyperandrogenism, in combination with up-regulation of AR expression, also leads to antiproliferative effects in glandular epithelium by suppressing oestrogen-dependent glandular mitosis despite the presence of oestrogen [122]. Thus, this effect delays endometrial proliferation and differentiation that is vital for endometrial receptivity.

**Figure 2:**
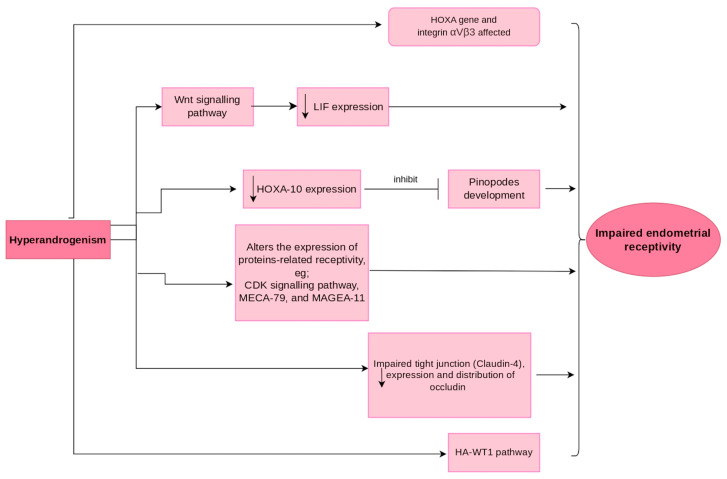
Possible mechanisms affecting the endometrial receptivity related to hyperandrogenism.

Particularly in PCOS patients, hyperandrogenism also increases the expression of the androgen receptor (AR) and the AR co-regulatory protein MAGEA11 in endometrial tissue, leading to a delay in endometrial decidualisation due to impaired differentiation of endometrial stromal cells [123]. As a result, the critical timing process of embryo implantation is disrupted, ultimately affecting human reproduction. Similarly, hyperandrogenism has been found to decrease the expression of αvβ3-integrin, E-cadherin, and mucin-1 (commonly referred to as receptivity markers) in the uterus during the receptivity period, thereby impairing successful embryo implantation and the establishment of pregnancy [124]. Hyperandrogenism also impairs endometrial receptivity by affecting intercellular tight junctions. In addition, the expression and distribution of claudin-4, a key protein component of endometrial tight junctions, and occludin were reduced in hyperandrogenism [125]. This leads to a porous tight junction that allows fluid movement through paracellular pathways which, in turn, can disrupt the implantation process. Figure 2 summarises the mechanisms involved in endometrial receptivity related to hyperandrogenism.

Furthermore, the combination of hyperandrogenism and insulin resistance activates ferroptosis due to decreased levels of glutathione peroxidase 4 (GPX4) and glutathione, increased glutathione + glutathione disulfide and malondialdehyde, aberrant expression of ferroptosis-associated genes (Acsl4, Tfrc, Slc7a11, and Gclc), increased iron deposition, and activated ERK/p38/JNK phosphorylation in the gravid uterus and placenta [126]. This process leads to a higher risk of early miscarriage. 

## 6. Impact of Uterine Anomalies in the Endometrial Molecular Expressions Affecting Endometrial Receptivity

Besides hyperandrogenism, there are many other causes that affect the receptivity of the endometrium and lead to infertility. Of all the causes, polyps, adenomyosis, and leiomyomas are associated with an increased likelihood of abnormal molecular expression of the endometrium, which is thought to interfere with implantation and early embryonic development [127].

Common benign uterine tumours, such as uterine myomas, are also called leiomyomas, fibroids, fibromyomas, fibroleiomyomas, and leiomyofibromas [128]. A primary cause for these tumours is a point mutation of the mediator complex subunit 12 (MED12) gene or the high-mobility group AT-hook2 (HMGA2) gene of a single leiomyoma cell [127]. A mutated MED12 gene resulted in inhibition of β-catenin transactivation in response to WNT signalling. TGF-β3 level is elevated when WNT/β-catenin pathway is activated. TGF-β3 is thought to mediate the production of BMP-2, which is responsible for mediating HOXA-10 [127]. A previous study demonstrated that HOXA-10 and LIF are decreased in submucosal leiomyomas, resulting in impaired decidualisation and reduced implantation success [129].

Adenomyosis is an ectopic, benign endometrial stroma and glands resides in the myometrium [130]. Adenomyosis impairs the receptivity of the endometrium, leading to poor outcomes, particularly with assisted reproductive technology. It has been hypothesised that adenomyosis may lead to impaired decidualisation due to abnormal concentrations of implantation markers, ultimately negatively affecting endometrial receptivity. It has also been suggested that the anatomical structure and function of the myometrium in adenomyosis alters normal endometrial peristalsis and impairs sperm motility, resulting in poor endometrial receptivity. LIF and FOXO-1A, which are important molecules in decidualisation, have been found to be down-regulated in adenomyosis patients. In addition, HOXA-10 was found to be down-regulated in women with adenomyosis, as well as in experimental adenomyosis in mouse models. Elevated levels of some inflammatory markers, such as IL -6, NK cells, macrophages and other cytokines, were found in adenomyotic tissue. Proteins such as β-catenin and L-selectin were also found to be increased in adenomyotic tissue [127].

A polyp is an abnormal growth typically consisting of fibrous tissue, the endometrium, stroma, and blood vessels. They can occur as either sessile or pedunculated forms and are present throughout the uterine cavity in varying sizes and numbers [131]. Approximately 32% of infertile women have polyps and two studies showed that the rate of artificial intrauterine insemination (IUI) improved after hysteroscopic polypectomy [132]. The possible mechanisms linking polyps to infertility are the release of chemicals or mechanical interference that may affect sperm motility or embryo implantation. Previous studies have shown that inflammatory markers, glycodelin, and aromatase are increased in patients with endometrial polyps and levels of HOXA-10 and HOXA-11, which are responsible for endometrial receptivity, are decreased [127].

## 7. Conclusions

In summary, the relationship between hyperandrogenism and endometrial receptivity in women without PCOS has not been adequately investigated. However, given the increased risk of both conditions in women with PCOS, it is likely that these associations are important. Further studies are needed to understand the mechanisms underlying this association and to develop interventions that may improve endometrial health in these women, particularly those of childbearing potential. Furthermore, the emphasis on endometrial function in hyperandrogenism-related infertility as well as uterine anomaly-related infertility may be more realistic given the widespread use of assisted reproductive technology. Overall, these findings highlight the importance of identifying and treating all factors contributing to the development of hyperandrogenism and endometrial receptivity, as this can improve health outcomes for women.

## Figures and Tables

**Table 1:** Methods used to determine endometrial receptivity and the associated parameters for receptive endometrium.

Endometrial Receptivity Marker	Receptive Endometrium	Less Receptive Endometrium	Accuracy	References
Endometrial thickness	>7 mm	<7 mm	Sensitivity: 99% Specificity: 3%	[88]
Endometrial volume	>2 mL	<2 mL	Sensitivity: 93% Specificity: 7%	[88]
Endometrial pattern	Presence of triple line pattern	Absence of triple line pattern	Sensitivity: 87% Specificity: 15%	[88]
Endometrial blood flow	Presence of flow	Absence of flow	Sensitivity: 100% Specificity: 8%	[88]
Endometrial contractions	Contractions absent	Contractions present	Sensitivity: 7% Specificity: 94%	[88]
Endometrial receptivity array (ERA)	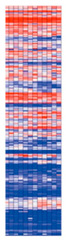	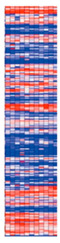 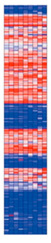	Insufficient data available	[88,108]

Transcriptomic analysis using heatmap analysis of Endometrial Receptivity Array (ERA) gene expression profiles utilising Babelomics platform.

## Data Availability

Not applicable.

## References

[1] Starc A., Trampuš M., Pavan Jukić D., Grgas-Bile C., Jukić T., Polona Mivšek A. (2019). Infertility and sexual dysfunctions: A systematic literature review. Acta Clin. Croat..

[2] Carson S.A., Kallen A.N. (2021). Diagnosis and management of infertility: A review. JAMA.

[3] Wang C., Wen Y.-X., Mai Q.-Y. (2022). Impact of metabolic disorders on endometrial receptivity in patients with polycystic ovary syndrome. Exp. Ther. Med..

[4] Horton R., Tait J. (1966). Androstenedione production and interconversion rates measured in peripheral blood and studies on the possible site of its conversion to testosterone. J. Clin. Investig..

[5] Azzouni F., Godoy A., Li Y., Mohler J. (2011). The 5 alpha-reductase isozyme family: A review of basic biology and their role in human diseases. Adv. Urol..

[6] Rosenfeld R., Hellman L., Roffwarg H., Weitzman E.D., Fukushima D.K., Gallagher T. (1971). Dehydroisoandrosterone is secreted episodically and synchronously with cortisol by normal man. J. Clin. Endocrinol. Metab..

[7] Marques P., Skorupskaite K., Rozario K.S., Anderson R.A., George J.T. (2022). Physiology of GNRH and gonadotropin secretion. Endotext [Internet].

[8] Iwasa T., Matsuzaki T., Yano K., Yanagihara R., Mayila Y., Irahara M. (2018). The effects of chronic testosterone administration on hypothalamic gonadotropin-releasing hormone regulatory factors (Kiss1, NKB, pDyn and RFRP) and their receptors in female rats. Gynecol. Endocrinol..

[9] Auchus R.J. (2004). The backdoor pathway to dihydrotestosterone. Trends Endocrinol. Metab..

[10] Bird C.E., Green R.N., Clark A.F. (1969). Effect of the administration of estrogen on the disappearance of 3H-testosterone in the plasma of human subjects. J. Clin. Endocrinol. Metab..

[11] Southren A.L., Gordon G.G., Tochimoto S., Krikun E., Krieger D., Jacobson M., Kuntzman R. (1969). Effect of N-phenylbarbital (phetharbital) on the metabolism of testosterone and cortisol in man. J. Clin. Endocrinol. Metab..

[12] Gordon G.G., Southren A.L., Tochimoto S., Rand J.J., Olivo J. (1969). Effect of hyperthyroidism and hypothyroidism on the metabolism of testosterone and androstenedione in man. J. Clin. Endocrinol. Metab..

[13] Southren A.L., Gordon G.G., Tochimoto S. (1968). Further study of factors affecting the metabolic clearance rate of testosterone in man. J. Clin. Endocrinol. Metab..

[14] Coviello A.D., Lakshman K., Mazer N.A., Bhasin S. (2006). Differences in the apparent metabolic clearance rate of testosterone in young and older men with gonadotropin suppression receiving graded doses of testosterone. J. Clin. Endocrinol. Metab..

[15] Cordera F., Grant C., Van Heerden J., Thompson G., Young W. (2003). Androgen-secreting adrenal tumors. Surgery.

[16] Critchley H.O., Saunders P.T. (2009). Hormone receptor dynamics in a receptive human endometrium. Reprod. Sci..

[17] Marshall E., Lowrey J., MacPherson S., Maybin J.A., Collins F., Critchley H.O., Saunders P.T. (2011). In silico analysis identifies a novel role for androgens in the regulation of human endometrial apoptosis. J. Clin. Endocrinol. Metab..

[18] Giudice L.C. (2006). Endometrium in PCOS: Implantation and predisposition to endocrine CA. Best Pract. Res. Clin. Endocrinol. Metab..

[19] Milne S.A., Henderson T.A., Kelly R.W., Saunders P.T., Baird D.T., Critchley H.O. (2005). Leukocyte populations and steroid receptor expression in human first-trimester decidua; regulation by antiprogestin and prostaglandin E analog. J. Clin. Endocrinol. Metab..

[20] Cloke B., Christian M. (2012). The role of androgens and the androgen receptor in cycling endometrium. Mol. Cell. Endocrinol..

[21] Zhang Y., Hu M., Yang F., Zhang Y., Ma S., Zhang D., Wang X., Sferruzzi-Perri A.N., Wu X., Brännström M. (2021). Increased uterine androgen receptor protein abundance results in implantation and mitochondrial defects in pregnant rats with hyperandrogenism and insulin resistance. J. Mol. Med..

[22] Azziz R., Carmina E., Sawaya M.E. (2000). Idiopathic hirsutism. Endocr. Rev..

[23] Luque-Ramírez M., F Escobar-Morreale H. (2016). Adrenal hyperandrogenism and polycystic ovary syndrome. Curr. Pharm. Des..

[24] Carmina E. (2006). Ovarian and adrenal hyperandrogenism. Ann. N. Y. Acad. Sci..

[25] Baptiste C.G., Battista M.-C., Trottier A., Baillargeon J.-P. (2010). Insulin and hyperandrogenism in women with polycystic ovary syndrome. J. Steroid Biochem. Mol. Biol..

[26] Barontini M., García-Rudaz M.C., Veldhuis J.D. (2001). Mechanisms of hypothalamic-pituitary-gonadal disruption in polycystic ovarian syndrome. Arch. Med. Res..

[27] Baskind N.E., Balen A.H. (2016). Hypothalamic–pituitary, ovarian and adrenal contributions to polycystic ovary syndrome. Best Pract. Res. Clin. Obstet. Gynaecol..

[28] Pretorius E., Arlt W., Storbeck K.-H. (2017). A new dawn for androgens: Novel lessons from 11-oxygenated C19 steroids. Mol. Cell. Endocrinol..

[29] Witchel S.F., Oberfield S.E., Peña A.S. (2019). Polycystic ovary syndrome: Pathophysiology, presentation, and treatment with emphasis on adolescent girls. J. Endocr. Soc..

[30] Gonzalez F. (1997). Adrenal involvement in polycystic ovary syndrome. Semin. Reprod. Endocrinol..

[31] Kostakis E.K., Gkioni L.N., Macut D., Mastorakos G. (2019). Androgens in menopausal women: Not only polycystic ovary syndrome. Hyperandrogenism Women.

[32] Morton N.M., Seckl J.R. (2008). 11β-hydroxysteroid dehydrogenase type 1 and obesity. Obes. Metab..

[33] Sandeep T.C., Walker B.R. (2001). Pathophysiology of modulation of local glucocorticoid levels by 11β-hydroxysteroid dehydrogenases. Trends Endocrinol. Metab..

[34] Ding H., Zhang J., Zhang F., Zhang S., Chen X., Liang W., Xie Q. (2021). Resistance to the insulin and elevated level of androgen: A major cause of polycystic ovary syndrome. Front. Endocrinol..

[35] Holte J., Bergh T., Gennarelli G., Wide L. (1994). The independent effects of polycystic ovary syndrome and obesity on serum concentrations of gonadotrophins and sex steroids in premenopausal women. Clin. Endocrinol..

[36] Azziz R., Carmina E., Dewailly D., Diamanti-Kandarakis E., Escobar-Morreale H.F., Futterweit W., Janssen O.E., Legro R.S., Norman R.J., Taylor A.E. (2009). The Androgen Excess and PCOS Society criteria for the polycystic ovary syndrome: The complete task force report. Fertil. Steril..

[37] Goodarzi M.O., Carmina E., Azziz R. (2015). Dhea, dheas and pcos. J. Steroid Biochem. Mol. Biol..

[38] Matheson E.M., Bain J. (2019). Hirsutism in women. Am. Fam. Physician.

[39] Macut D., Ilić D., Jovanović A.M., Bjekić-Macut J. (2019). Androgen-secreting ovarian tumors. Hyperandrogenism Women.

[40] Witchel S.F. (2017). Congenital adrenal hyperplasia. J. Pediatr. Adolesc. Gynecol..

[41] Parsa A.A., New M.I. (2017). Steroid 21-hydroxylase deficiency in congenital adrenal hyperplasia. J. Steroid Biochem. Mol. Biol..

[42] Nandagopal R., Sinaii N., Avila N.A., Van Ryzin C., Chen W., Finkielstain G.P., Mehta S.P., McDonnell N.B., Merke D.P. (2011). Phenotypic profiling of parents with cryptic nonclassic congenital adrenal hyperplasia: Findings in 145 unrelated families. Eur. J. Endocrinol..

[43] Pignatelli D., Pereira S.S., Pasquali R. (2019). Androgens in congenital adrenal hyperplasia. Hyperandrogenism Women.

[44] Pall M., Azziz R., Beires J., Pignatelli D. (2010). The phenotype of hirsute women: A comparison of polycystic ovary syndrome and 21-hydroxylase–deficient nonclassic adrenal hyperplasia. Fertil. Steril..

[45] Raff H., Sharma S.T., Nieman L.K. (2014). Physiological basis for the etiology, diagnosis, and treatment of adrenal disorders: Cushing’s syndrome, adrenal insufficiency, and congenital adrenal hyperplasia. Compr. Physiol..

[46] Escobar-Morreale H., Carmina E., Dewailly D., Gambineri A., Kelestimur F., Moghetti P., Pugeat M., Qiao J., Wijeyaratne C., Witchel S. (2012). Epidemiology, diagnosis and management of hirsutism: A consensus statement by the Androgen Excess and Polycystic Ovary Syndrome Society. Hum. Reprod. Update.

[47] Binay C., Simsek E., Cilingir O., Yuksel Z., Kutlay O., Artan S. (2014). Prevalence of nonclassic congenital adrenal hyperplasia in Turkish children presenting with premature pubarche, hirsutism, or oligomenorrhoea. Int. J. Endocrinol..

[48] Armengaud J.-B., Charkaluk M.-L., Trivin C., Tardy V., Bréart G., Brauner R., Chalumeau M. (2009). Precocious pubarche: Distinguishing late-onset congenital adrenal hyperplasia from premature adrenarche. J. Clin. Endocrinol. Metab..

[49] Azziz R., Waggoner W.T., Ochoa T., Knochenhauer E.S., Boots L.R. (1998). Idiopathic hirsutism: An uncommon cause of hirsutism in Alabama. Fertil. Steril..

[50] Azziz R., Sanchez L., Knochenhauer E., Moran C., Lazenby J., Stephens K., Taylor K., Boots L. (2004). Androgen excess in women: Experience with over 1000 consecutive patients. J. Clin. Endocrinol. Metab..

[51] Bidet M., Bellanne-Chantelot C., Galand-Portier M.-B., Golmard J.-L., Tardy V., Morel Y., Clauin S., Coussieu C., Boudou P., Mowzowicz I. (2010). Fertility in women with nonclassical congenital adrenal hyperplasia due to 21-hydroxylase deficiency. J. Clin. Endocrinol. Metab..

[52] Lobo R.A., Goebelsmann U. (1980). Adult manifestation of congenital adrenal hyperplasia due to incomplete 21-hydroxylase deficiency mimicking polycystic ovarian disease. Am. J. Obstet. Gynecol..

[53] Escobar-Morreale H.F., Sanchon R., San Millán J.L. (2008). A prospective study of the prevalence of nonclassical congenital adrenal hyperplasia among women presenting with hyperandrogenic symptoms and signs. J. Clin. Endocrinol. Metab..

[54] Pignatelli D. (2013). Non-classic adrenal hyperplasia due to the deficiency of 21-hydroxylase and its relation to polycystic ovarian syndrome. Polycystic Ovary Syndr..

[55] Roland A.V., Moenter S.M. (2014). Reproductive neuroendocrine dysfunction in polycystic ovary syndrome: Insight from animal models. Front. Neuroendocrinol..

[56] Pache T., Chadha S., Gooren L., Hop W., Jaarsma K., Dommerholt H., Fauser B. (1991). Ovarian morphology in long-term androgen-treated female to male transsexuals. A human model for the study of polycystic ovarian syndrome?. Histopathology.

[57] Lucis O., Hobkirk R., Hollenberg C., MacDonald S., Blahey P. (1966). Polycystic ovaries associated with congenital adrenal hyperplasia. Can. Med. Assoc. J..

[58] Sen A., Hammes S.R. (2010). Granulosa cell-specific androgen receptors are critical regulators of ovarian development and function. Mol. Endocrinol..

[59] Walters K.A. (2015). Role of androgens in normal and pathological ovarian function. Reproduction.

[60] Walters K., Handelsman D. (2018). Role of androgens in the ovary. Mol. Cell. Endocrinol..

[61] Gleicher N., Weghofer A., Barad D.H. (2011). The role of androgens in follicle maturation and ovulation induction: Friend or foe of infertility treatment?. Reprod. Biol. Endocrinol..

[62] Lebbe M., Woodruff T. (2013). Involvement of androgens in ovarian health and disease. Mol. Hum. Reprod..

[63] Mulaikal R.M., Migeon C.J., Rock J.A. (1987). Fertility rates in female patients with congenital adrenal hyperplasia due to 21-hydroxylase deficiency. N. Engl. J. Med..

[64] Anwar S., Anwar A. (2016). Infertility: A review on causes, treatment and management. Womens Health Gynecol..

[65] Dédjan A.H., Chadli A., El Aziz S., Farouqi A. (2015). Hyperandrogenism-Insulin Resistance-Acanthosis Nigricans Syndrome. Case Rep. Endocrinol..

[66] Elmer K.B., George R.M. (2001). HAIR-AN syndrome: A multisystem challenge. Am. Fam. Physician.

[67] Esperanza L.E., Fenske N.A. (1996). Hyperandrogenism, insulin resistance, and acanthosis nigricans (HAIR-AN) syndrome: Spontaneous remission in a 15-year-old girl. J. Am. Acad. Dermatol..

[68] Brady M.F., Rawla P. (2022). Acanthosis Nigricans. StatPearls.

[69] Rager K.M., Omar H.A. (2006). Androgen excess disorders in women: The severe insulin-resistant hyperandrogenic syndrome, HAIR-AN. Sci. World J..

[70] Hermanns-Lê T., Scheen A., Piérard G.E. (2004). Acanthosis nigricans associated with insulin resistance. Am. J. Clin. Dermatol..

[71] Ferriman D., Gallwey J. (1961). Clinical assessment of body hair growth in women. J. Clin. Endocrinol. Metab..

[72] Lessey B.A., Young S.L. (2019). What exactly is endometrial receptivity?. Fertil. Steril..

[73] Rock J., Bartlett M.K. (1937). Biopsy studies of human endometrium: Criteria of dating and information about amenorrhea, menorrhagia and time of ovulation. J. Am. Med. Assoc..

[74] Tan J., Paria B.C., Dey S.K., Das S.K. (1999). Differential uterine expression of estrogen and progesterone receptors correlates with uterine preparation for implantation and decidualization in the mouse. Endocrinology.

[75] Gibson D.A., Simitsidellis I., Cousins F.L., Critchley H.O., Saunders P.T. (2016). Intracrine androgens enhance decidualization and modulate expression of human endometrial receptivity genes. Sci. Rep..

[76] Piltonen T.T. (2016). Polycystic ovary syndrome: Endometrial markers. Best Pract. Res. Clin. Obstet. Gynaecol..

[77] Gellersen B., Brosens I.A., Brosens J.J. (2007). Decidualization of the human endometrium: Mechanisms, functions, and clinical perspectives. Semin. Reprod. Med..

[78] Governini L., Luongo F.P., Haxhiu A., Piomboni P., Luddi A. (2021). Main actors behind the endometrial receptivity and successful implantation. Tissue Cell.

[79] Usadi R.S., Murray M.J., Bagnell R.C., Fritz M.A., Kowalik A.I., Meyer W.R., Lessey B.A. (2003). Temporal and morphologic characteristics of pinopod expression across the secretory phase of the endometrial cycle in normally cycling women with proven fertility. Fertil. Steril..

[80] Fitzgerald H.C., Salamonsen L.A., Rombauts L.J., Vollenhoven B.J., Edgell T.A. (2016). The proliferative phase underpins endometrial development: Altered cytokine profiles in uterine lavage fluid of women with idiopathic infertility. Cytokine.

[81] Zhao Y., Garcia J., Kolp L., Cheadle C., Rodriguez A., Vlahos N.F. (2010). The impact of luteal phase support on gene expression of extracellular matrix protein and adhesion molecules in the human endometrium during the window of implantation following controlled ovarian stimulation with a GnRH antagonist protocol. Fertil. Steril..

[82] Robertshaw I., Bian F., Das S. (2016). Mechanisms of uterine estrogen signaling during early pregnancy in mice: An update. J. Mol. Endocrinol..

[83] Wang X., Wu S.-P., DeMayo F.J. (2017). Hormone dependent uterine epithelial-stromal communication for pregnancy support. Placenta.

[84] Kodaman P.H., Taylor H.S. (2004). Hormonal regulation of implantation. Obstet. Gynecol. Clin..

[85] Bulmer J.N., Morrison L., Longfellow M., Ritson A., Pace D. (1991). Granulated lymphocytes in human endometrium: Histochemical and immunohistochemical studies. Hum. Reprod..

[86] Adams S., Gayer N., Hosie M., Murphy C. (2002). Human uterodomes (pinopods) do not display pinocytotic function. Hum. Reprod..

[87] Zhao J., Zhang Q., Li Y. (2012). The effect of endometrial thickness and pattern measured by ultrasonography on pregnancy outcomes during IVF-ET cycles. Reprod. Biol. Endocrinol..

[88] Craciunas L., Gallos I., Chu J., Bourne T., Quenby S., Brosens J.J., Coomarasamy A. (2019). Conventional and modern markers of endometrial receptivity: A systematic review and meta-analysis. Hum. Reprod. Update.

[89] Zollner U., Zollner K.-P., Specketer M.-T., Blissing S., Müller T., Steck T., Dietl J. (2003). Endometrial volume as assessed by three-dimensional ultrasound is a predictor of pregnancy outcome after in vitro fertilization and embryo transfer. Fertil. Steril..

[90] Wang L., Qiao J., Li R., Zhen X., Liu Z. (2010). Role of endometrial blood flow assessment with color Doppler energy in predicting pregnancy outcome of IVF-ET cycles. Reprod. Biol. Endocrinol..

[91] Chung C.H.S., Wong A.W.Y., Chan C.P.S., Saravelos S.H., Kong G.W.S., Cheung L.P., Chung J.P.W., Li T.C. (2017). The changing pattern of uterine contractions before and after fresh embryo transfer and its relation to clinical outcome. Reprod. Biomed. Online.

[92] Sharma S. (2014). Natural killer cells and regulatory T cells in early pregnancy loss. Int. J. Dev. Biol..

[93] Sauerbrun-Cutler M.T., Huber W.J., Krueger P.M., Sung C.J., Has P., Sharma S. (2021). Do endometrial natural killer and regulatory T cells differ in infertile and clinical pregnancy patients? An analysis in patients undergoing frozen embryo transfer cycles. Am. J. Reprod. Immunol..

[94] Fu B., Li X., Sun R., Tong X., Ling B., Tian Z., Wei H. (2013). Natural killer cells promote immune tolerance by regulating inflammatory TH17 cells at the human maternal–fetal interface. Proc. Natl. Acad. Sci. USA.

[95] Gaynor L.M., Colucci F. (2017). Uterine natural killer cells: Functional distinctions and influence on pregnancy in humans and mice. Front. Immunol..

[96] Díaz-Gimeno P., Horcajadas J.A., Martínez-Conejero J.A., Esteban F.J., Alamá P., Pellicer A., Simón C. (2011). A genomic diagnostic tool for human endometrial receptivity based on the transcriptomic signature. Fertil. Steril..

[97] Mirkin S., Nikas G., Hsiu J.-G., Díaz J., Oehninger S. (2004). Gene expression profiles and structural/functional features of the peri-implantation endometrium in natural and gonadotropin-stimulated cycles. J. Clin. Endocrinol. Metab..

[98] Díaz-Gimeno P., Ruiz-Alonso M., Sebastian-Leon P., Pellicer A., Valbuena D., Simón C. (2017). Window of implantation transcriptomic stratification reveals different endometrial subsignatures associated with live birth and biochemical pregnancy. Fertil. Steril..

[99] Ruiz-Alonso M., Blesa D., Díaz-Gimeno P., Gómez E., Fernández-Sánchez M., Carranza F., Carrera J., Vilella F., Pellicer A., Simón C. (2013). The endometrial receptivity array for diagnosis and personalized embryo transfer as a treatment for patients with repeated implantation failure. Fertil. Steril..

[100] Ruiz-Alonso M., Galindo N., Pellicer A., Simón C. (2014). What a difference two days make:“personalized” embryo transfer (pET) paradigm: A case report and pilot study. Hum. Reprod..

[101] Tan J., Kan A., Hitkari J., Taylor B., Tallon N., Warraich G., Yuzpe A., Nakhuda G. (2018). The role of the endometrial receptivity array (ERA) in patients who have failed euploid embryo transfers. J. Assist. Reprod. Genet..

[102] Hashimoto T., Koizumi M., Doshida M., Toya M., Sagara E., Oka N., Nakajo Y., Aono N., Igarashi H., Kyono K. (2017). Efficacy of the endometrial receptivity array for repeated implantation failure in Japan: A retrospective, two-centers study. Reprod. Med. Biol..

[103] Mahajan N. (2015). Endometrial receptivity array: Clinical application. J. Hum. Reprod. Sci..

[104] Ma L., Cao Y., Ma Y., Zhai J. (2021). Association between hyperandrogenism and adverse pregnancy outcomes in patients with different polycystic ovary syndrome phenotypes undergoing in vitro fertilization/intracytoplasmic sperm injection: A systematic review and meta-analysis. Gynecol. Endocrinol..

[105] Yang W., Yang R., Yang S., Li J., Tu B., Gao C., Wang Y. (2018). Infertile polycystic ovary syndrome patients undergoing in vitro fertilization with the gonadotropin-releasing hormone-antagonist protocol: Role of hyperandrogenism. Gynecol. Endocrinol..

[106] Pan D., Shi J., Zhou H., Li N., Qu P. (2018). Predictive value of basal androgen levels on ongoing pregnancy rates during in vitro fertilization cycles. Gynecol. Endocrinol..

[107] Lam P., Johnson I., Raine-Fenning N. (2009). Endometrial blood flow is impaired in women with polycystic ovarian syndrome who are clinically hyperandrogenic. Ultrasound Obstet. Gynecol. Off. J. Int. Soc. Ultrasound Obstet. Gynecol..

[108] Díaz-Gimeno P., Ruíz-Alonso M., Blesa D., Simón C. (2014). Transcriptomics of the human endometrium. Int. J. Dev. Biol..

[109] Jiang N.X., Li X.L. (2022). The Disorders of Endometrial Receptivity in PCOS and Its Mechanisms. Reprod. Sci..

[110] Mills L.J., Gutjahr-Gobell R.E., Zaroogian G.E., Horowitz D.B., Laws S.C. (2014). Modulation of aromatase activity as a mode of action for endocrine disrupting chemicals in a marine fish. Aquat. Toxicol..

[111] Bulun S.E., Sebastian S., Takayama K., Suzuki T., Sasano H., Shozu M. (2003). The human CYP19 (aromatase P450) gene: Update on physiologic roles and genomic organization of promoters. J. Steroid Biochem. Mol. Biol..

[112] Ma C.X., Adjei A.A., Salavaggione O.E., Coronel J., Pelleymounter L., Wang L., Eckloff B.W., Schaid D., Wieben E.D., Adjei A.A. (2005). Human aromatase: Gene resequencing and functional genomics. Cancer Res..

[113] Chen J., Shen S., Tan Y., Xia D., Xia Y., Cao Y., Wang W., Wu X., Wang H., Yi L. (2015). The correlation of aromatase activity and obesity in women with or without polycystic ovary syndrome. J. Ovarian Res..

[114] Gonzalez D., Thackeray H., Lewis P., Mantani A., Brook N., Ahuja K., Margara R., Joels L., White J., Conlan R.S. (2012). Loss of WT1 expression in the endometrium of infertile PCOS patients: A hyperandrogenic effect?. J. Clin. Endocrinol. Metab..

[115] Apparao K., Lovely L.P., Gui Y., Lininger R.A., Lessey B.A. (2002). Elevated endometrial androgen receptor expression in women with polycystic ovarian syndrome. Biol. Reprod..

[116] Cakmak H., Taylor H.S. (2011). Implantation failure: Molecular mechanisms and clinical treatment. Hum. Reprod. Update.

[117] Cermik D., Selam B., Taylor H.S. (2003). Regulation of HOXA-10 expression by testosterone in vitro and in the endometrium of patients with polycystic ovary syndrome. J. Clin. Endocrinol. Metab..

[118] Gregory C.W., Wilson E.M., Apparao K., Lininger R.A., Meyer W.R., Kowalik A., Fritz M.A., Lessey B.A. (2002). Steroid receptor coactivator expression throughout the menstrual cycle in normal and abnormal endometrium. J. Clin. Endocrinol. Metab..

[119] Senturk S., Celik O., Dalkilic S., Hatirnaz S., Celik N., Unlu C., Otlu B. (2020). Laparoscopic ovarian drilling improves endometrial Homeobox gene expression in PCOS. Reprod. Sci..

[120] Rahman T.U., Ullah K., Guo M.-X., Pan H.-T., Liu J., Ren J., Jin L.-Y., Zhou Y.-Z., Cheng Y., Sheng J.-Z. (2018). Androgen-induced alterations in endometrial proteins crucial in recurrent miscarriages. Oncotarget.

[121] Mokhtar M.H., Giribabu N., Salleh N. (2020). Testosterone decreases the number of implanting embryos, expression of pinopode and L-selectin ligand (MECA-79) in the endometrium of early pregnant rats. Int. J. Environ. Res. Public Health.

[122] Brenner R.M., Slayden O.D., Critchley H. (2002). Anti-proliferative effects of progesterone antagonists in the primate endometrium: A potential role for the androgen receptor. Reproduction.

[123] Younas K., Quintela M., Thomas S., Garcia-Parra J., Blake L., Whiteland H., Bunkheila A., Francis L.W., Margarit L., Gonzalez D. (2019). Delayed endometrial decidualisation in polycystic ovary syndrome; the role of AR-MAGEA11. J. Mol. Med..

[124] Mokhtar H.M., Giribabu N., Salleh N. (2018). Testosterone down-regulates expression of αVβ3-integrin, E-cadherin and mucin-1 during uterine receptivity period in rats. Sains Malays..

[125] Mokhtar M.H., Giribabu N., Salleh N. (2020). Testosterone reduces tight junction complexity and down-regulates expression of claudin-4 and occludin in the endometrium in ovariectomized, sex-steroid replacement rats. In Vivo.

[126] Zhang Y., Hu M., Jia W., Liu G., Zhang J., Wang B., Li J., Cui P., Li X., Lager S. (2020). Hyperandrogenism and insulin resistance modulate gravid uterine and placental ferroptosis in PCOS-like rats. J. Endocrinol..

[127] Munro M.G. (2019). Uterine polyps, adenomyosis, leiomyomas, and endometrial receptivity. Fertil. Steril..

[128] Vlahos N.F., Theodoridis T.D., Partsinevelos G.A. (2017). Myomas and adenomyosis: Impact on reproductive outcome. BioMed Res. Int..

[129] Lisiecki M., Paszkowski M., Woźniak S. (2017). Fertility impairment associated with uterine fibroids–a review of literature. Menopause Rev./Przegląd Menopauzalny.

[130] Antero M.F., Ayhan A., Segars J., Shih I.-M. (2020). Pathology and pathogenesis of adenomyosis. Semin. Reprod. Med..

[131] Nijkang N.P., Anderson L., Markham R., Manconi F. (2019). Endometrial polyps: Pathogenesis, sequelae, and treatment. SAGE Open Med..

[132] Bosteels J., Kasius J., Weyers S., Broekmans F.J., Mol B.W.J., D’Hooghe T.M. (2018). Hysteroscopy for treating subfertility associated with suspected major uterine cavity abnormalities Cochrane Database of Systematic Reviews. Cochrane Database Syst. Rev..

